# Supplementary material for: Personal Health Record for Personalizing Research and Care Trajectories: A Proof of Concept Pilot with Diet in Inflammatory Bowel Diseases
Source: J Pers Med. 2023 Mar 29;13(4):601. doi: 10.3390/jpm13040601 (PMC10144383; doi:10.3390/jpm13040601)

Mai+Science & Mai+Life Screenshots

Phaitality

Mai+Life: Benaderbaarheid

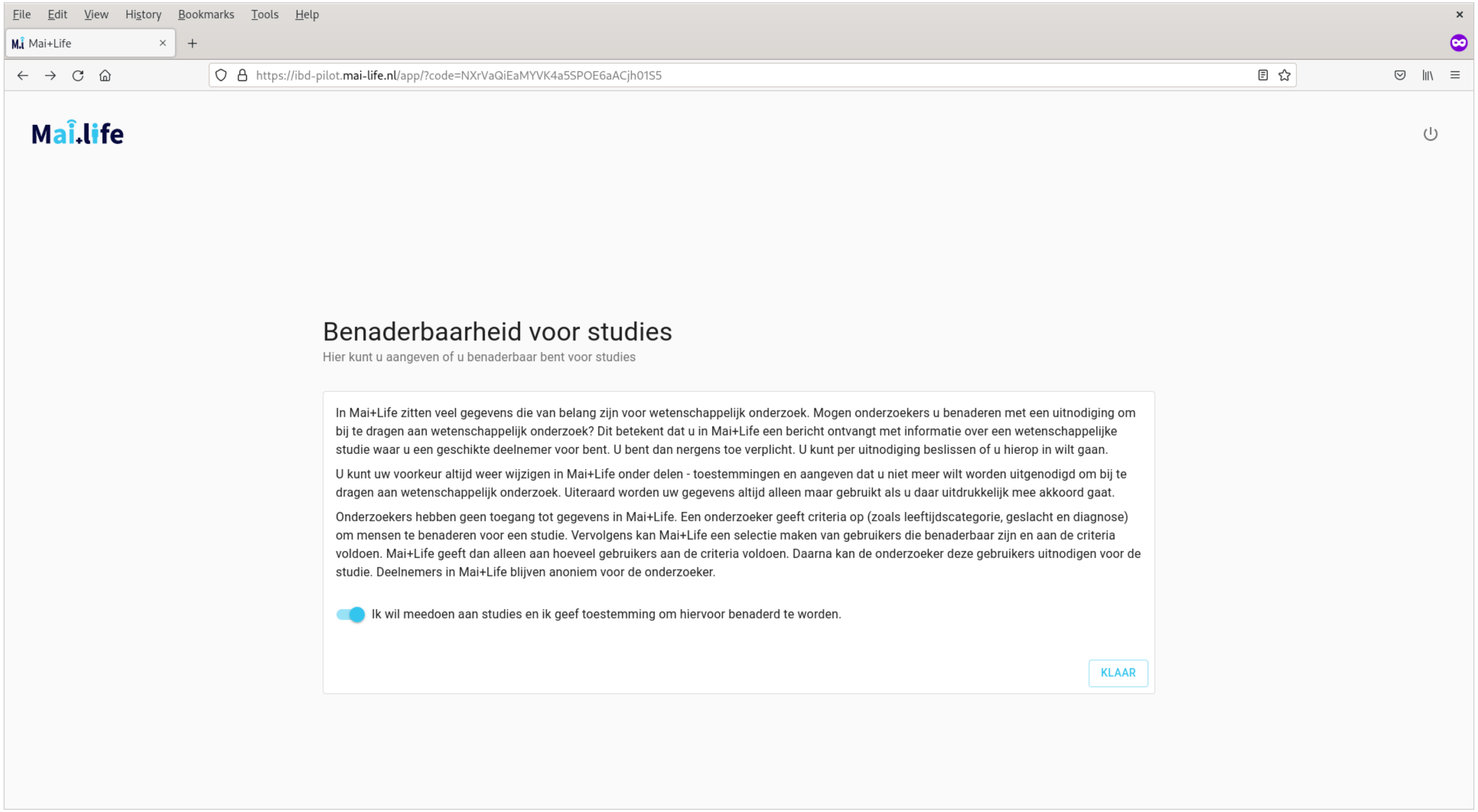

Mai+Life: Onderzoek

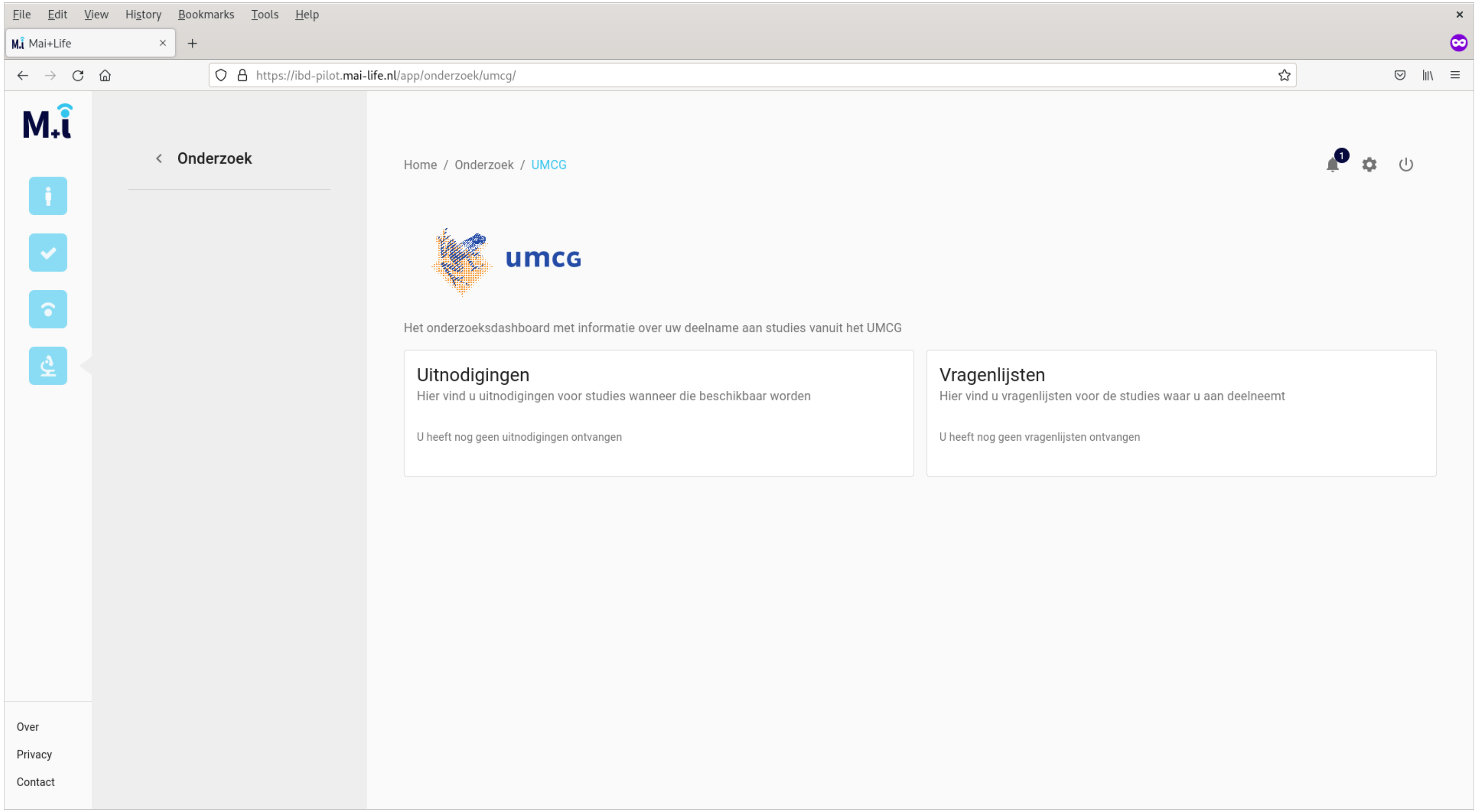

Mai+Science dashboard

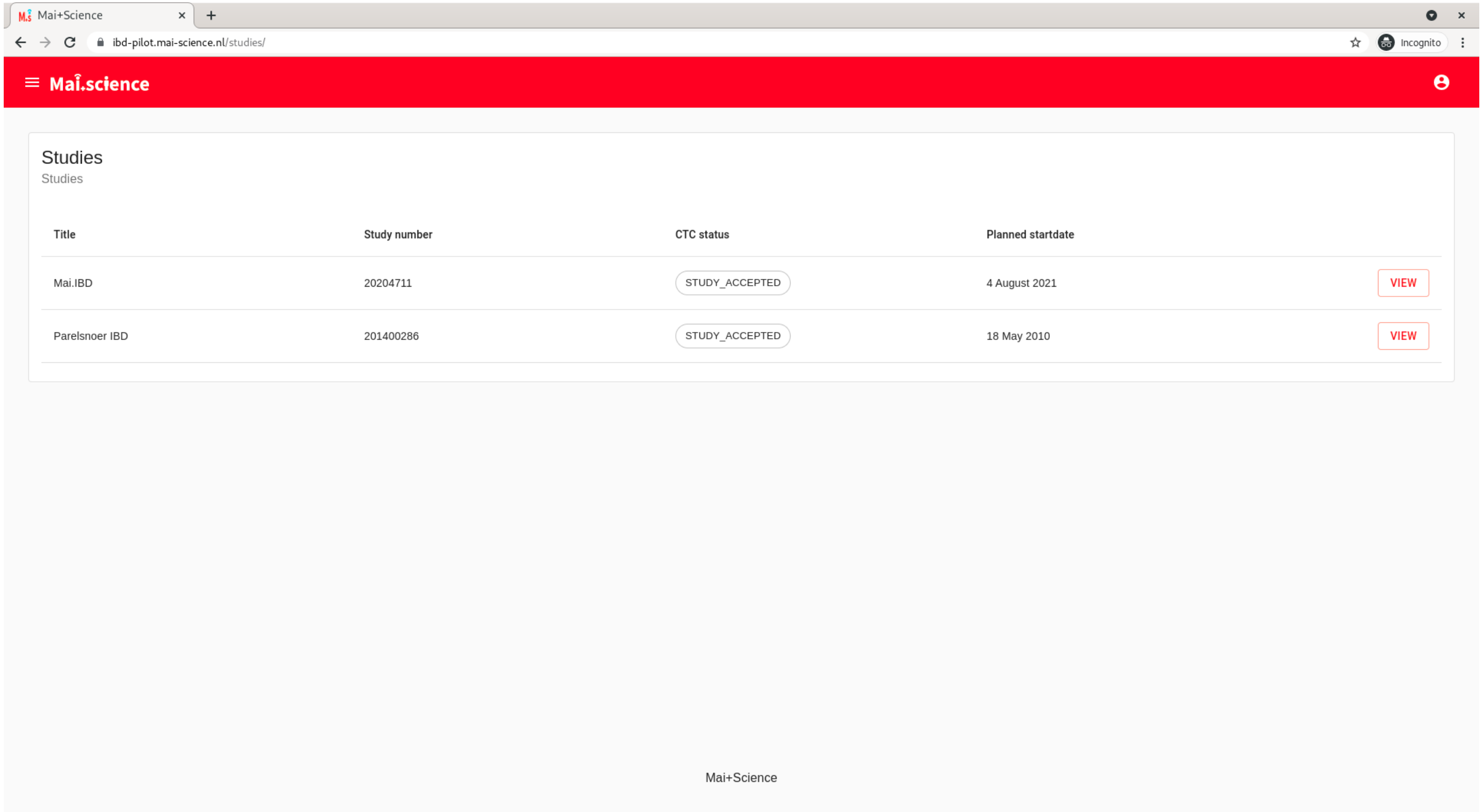

Mai+Science:Study Dashboard

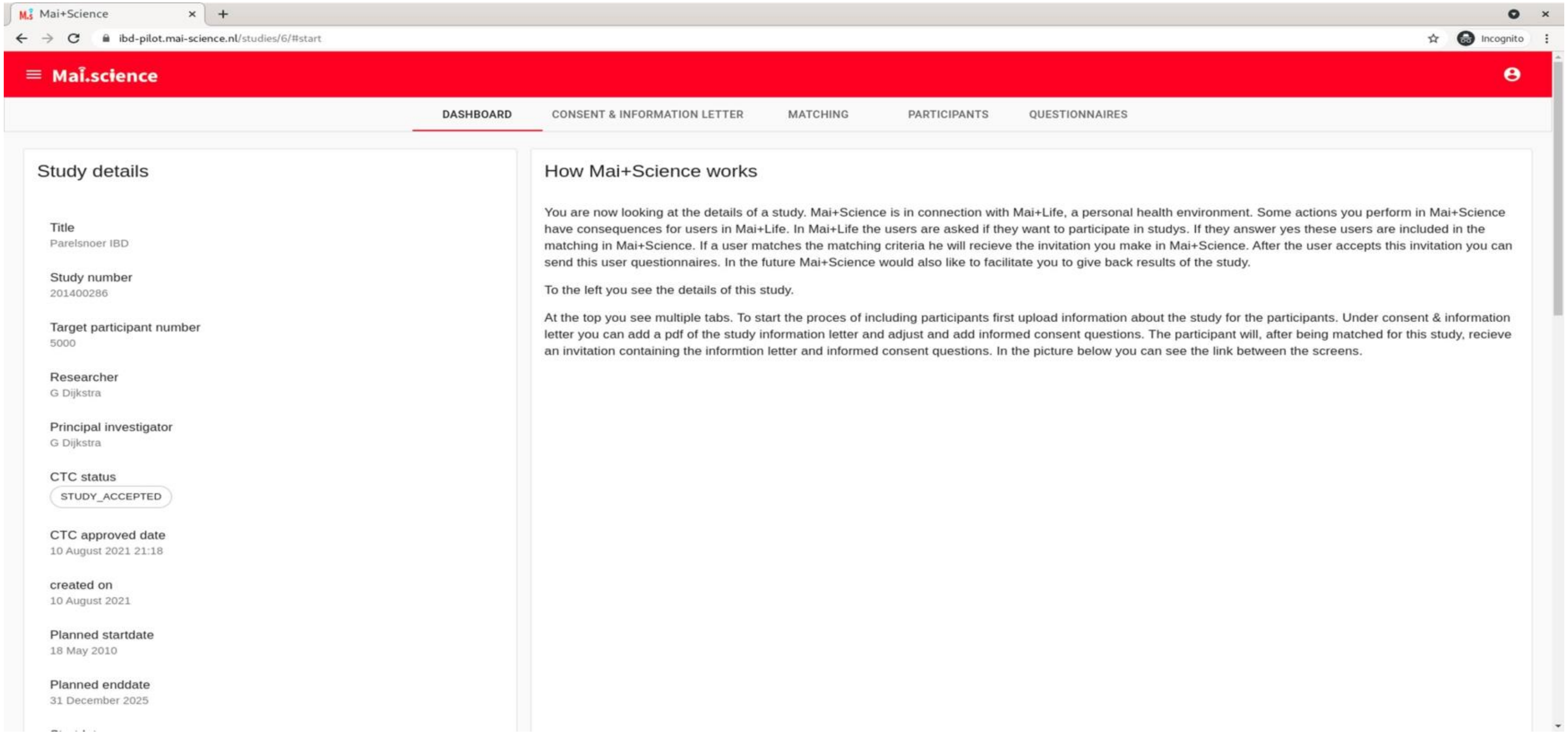

Mai+Science:Consent & Information Letter

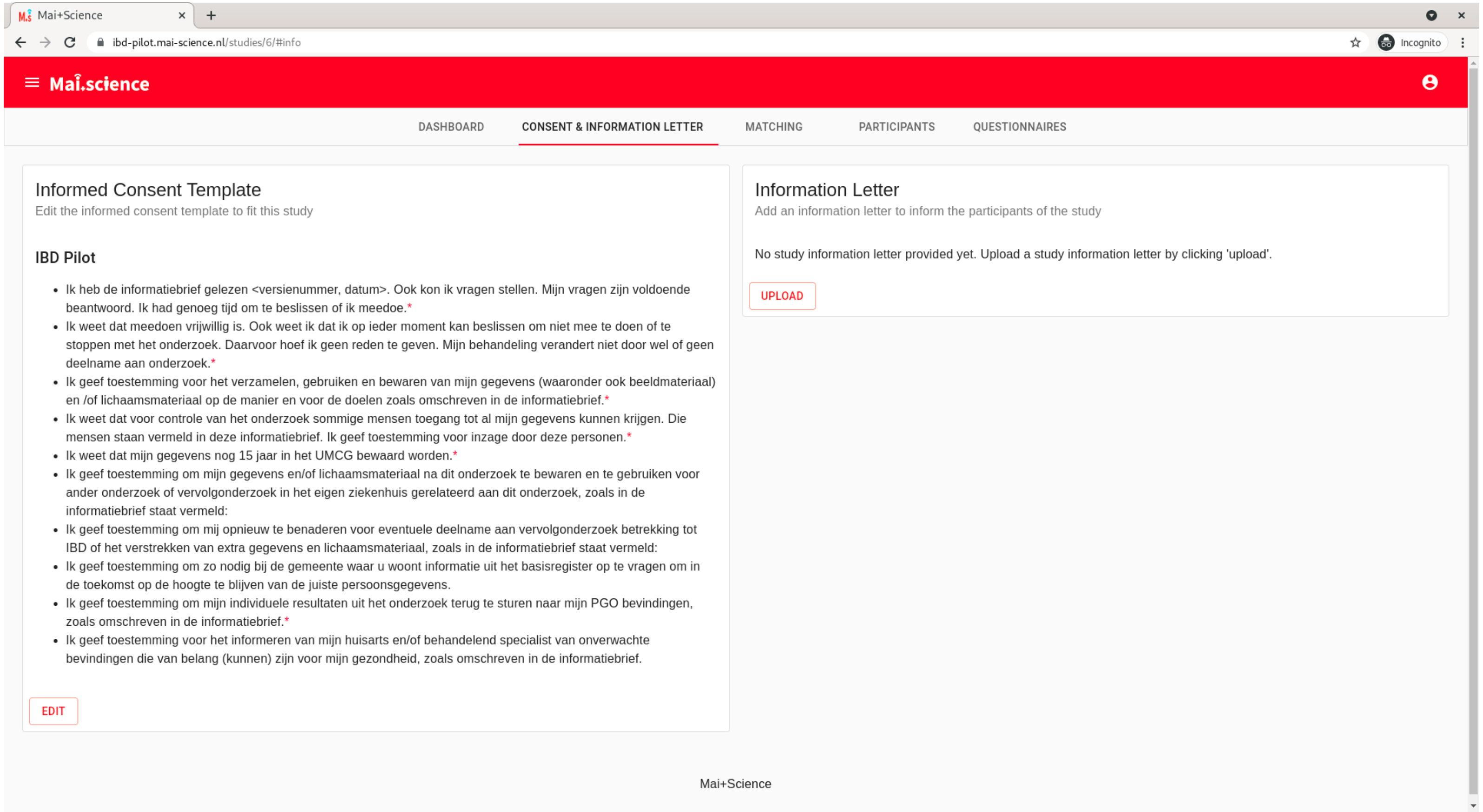

Mai+Science: Matching

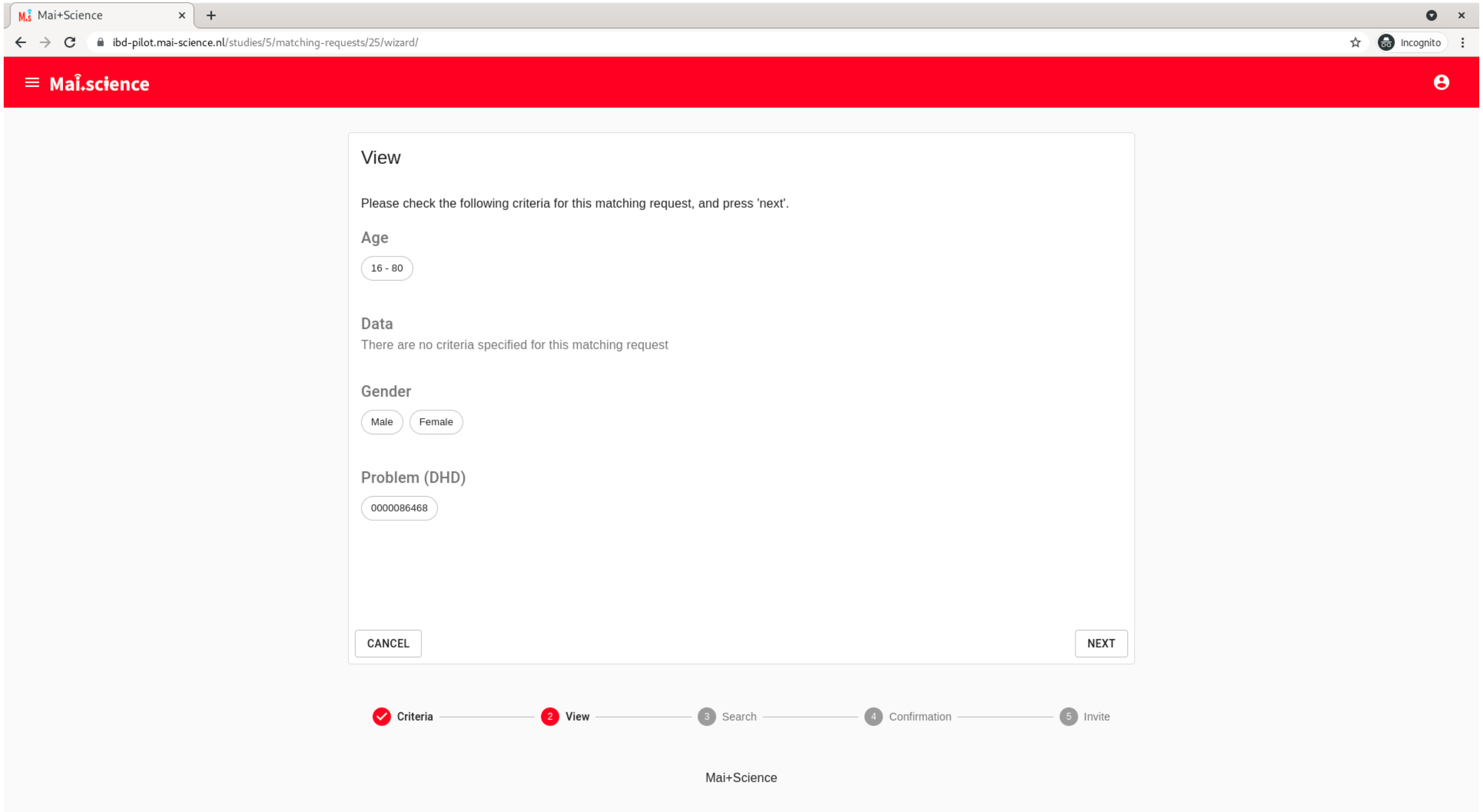

Mai+Life: Uitnodiging

File Edit View History Bookmarks Tools Help

Mai+Life

https://ibd-pilot.mai-life.nl/app/

Mai.life

U bent uitgenodigd voor een studie!

Klik op 'informatiebrief' om meer informatie over deze studie te kunnen lezen. Als u nu niet wilt beslissen of u aan deze studie deel wilt nemen, klik dan op 'negeren'. U kunt dan op een later tijdstip in Mai+Life alsnog de informatie over deze studie lezen en dan uw keuze maken. Als u wel deel wilt nemen aan de studie klik dan op 'ga verder'. U krijgt dan een aantal toestemmingsvragen te zien. U kunt na het bekijken van de toestemmingsvragen alsnog besluiten om niet deel te nemen aan de studie. Alleen als u akkoord gaat met alle verplichte toestemmingsvragen kan u deelnemen. Als u niet deel wilt nemen aan de studie, klik dan op 'afwijzen'. Op een later moment kunt u in Mai+Life ook na accepatie of afwijzing van deze uitnodiging uw keuze nog aanpassen.

INFORMATIEBRIEF

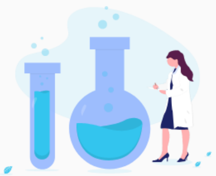

AFWIJZEN NEGEREN VOLGENDE

Mai+Life: Toestemming

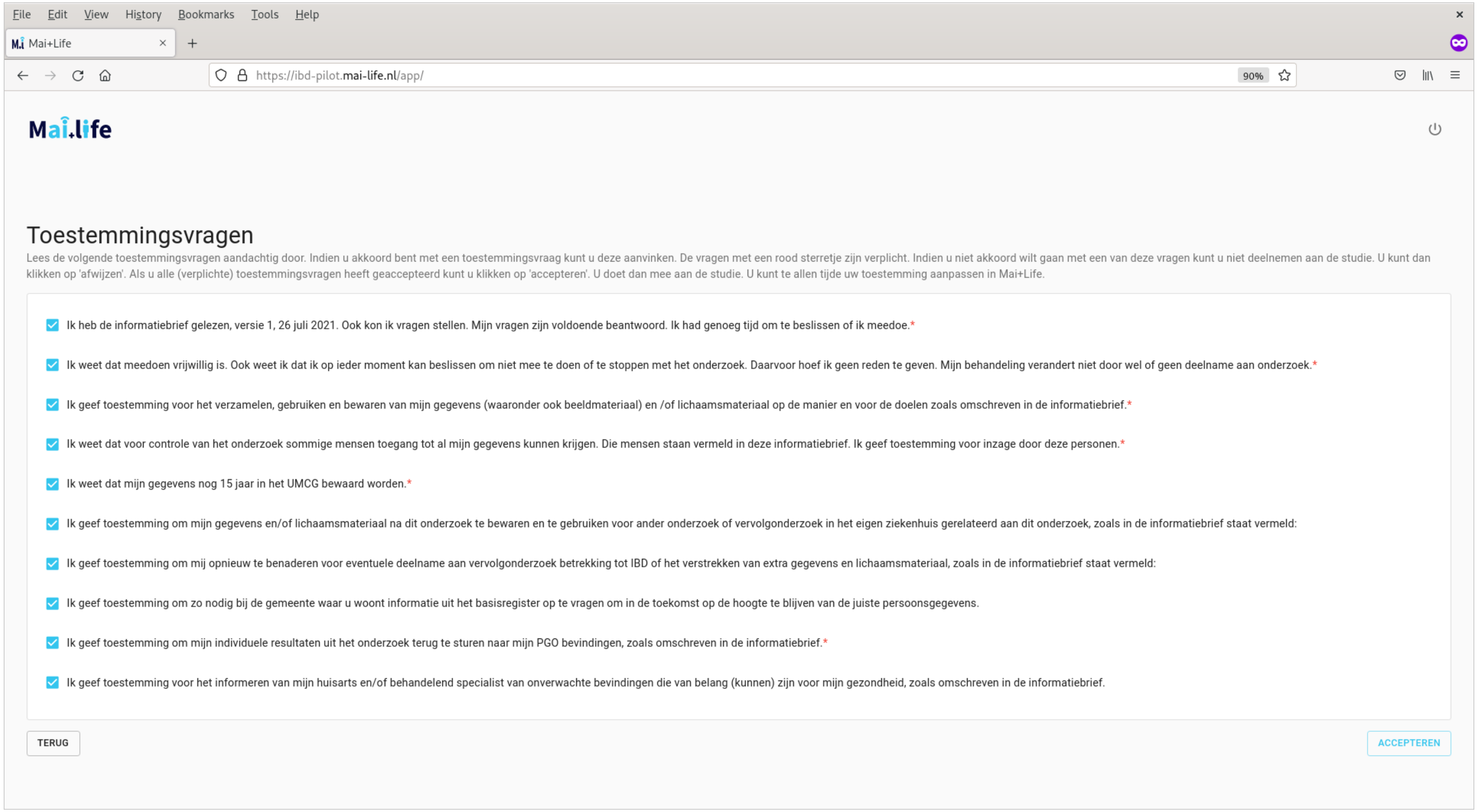

Mai+Science:Status participant

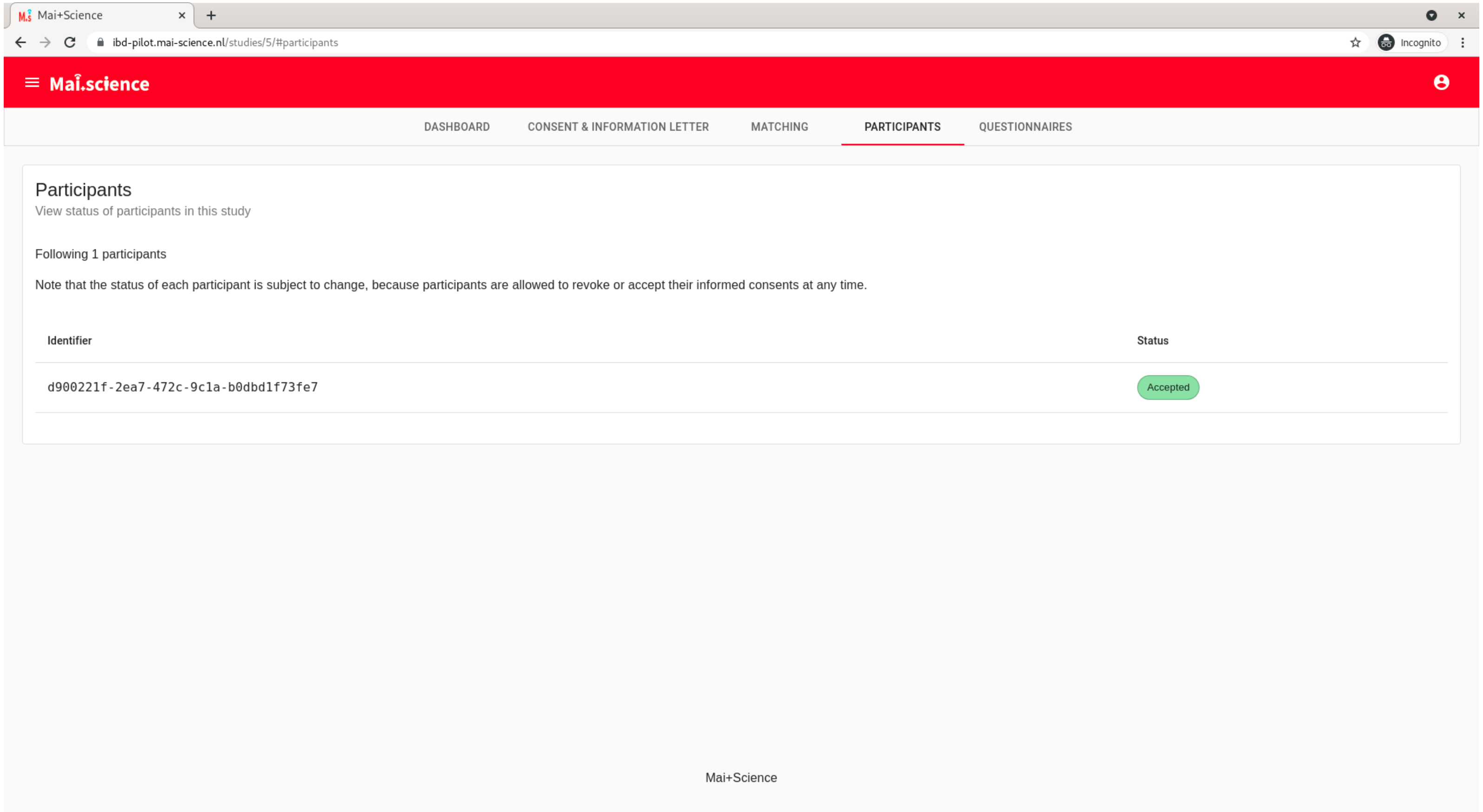

Mai+Science:Questionnaires

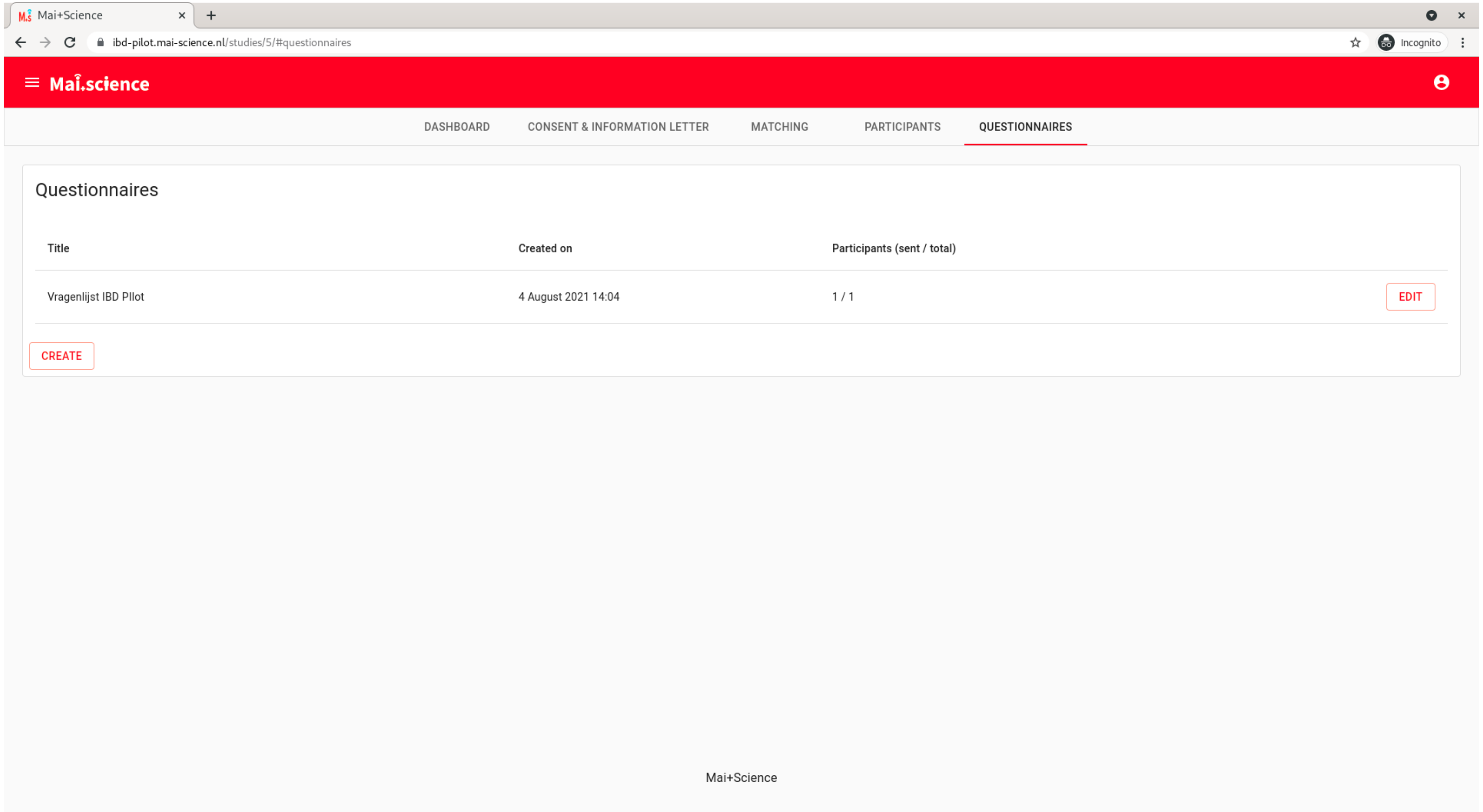

Mai+Life: Vragenlijsten

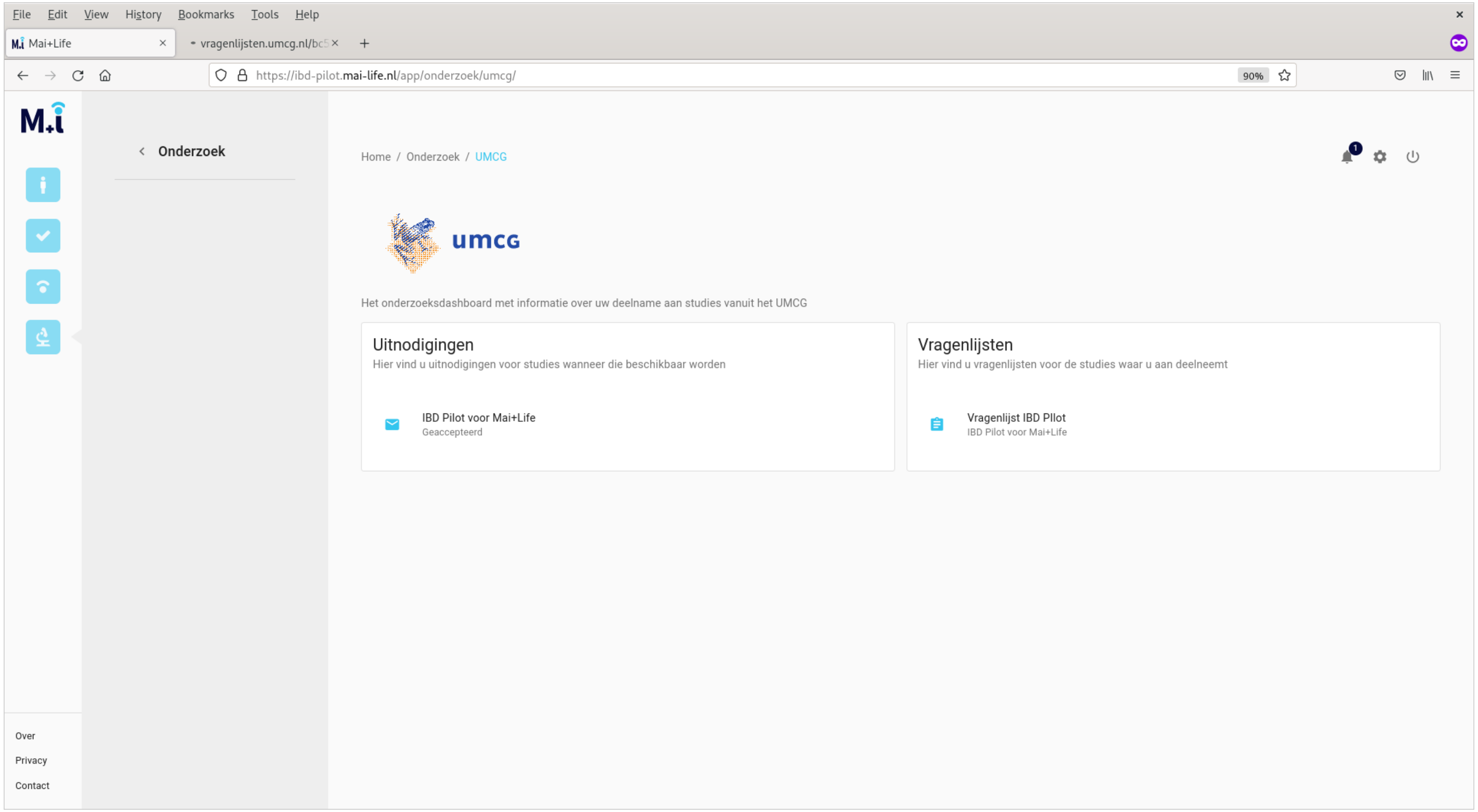

Supplement: Supplementary file 1 [file jpm-13-00601-s001.zip › Mai-Life-Science-Screenshots.pdf]
