# Supplementary material for: Personal Health Record for Personalizing Research and Care Trajectories: A Proof of Concept Pilot with Diet in Inflammatory Bowel Diseases
Source: J Pers Med. 2023 Mar 29;13(4):601. doi: 10.3390/jpm13040601 (PMC10144383; doi:10.3390/jpm13040601)
Supplement: Supplementary file 1 [file jpm-13-00601-s001.zip › PHR_IBDpilot_topicguide.pdf]

Topic guide: Current research participants' views and expectations on receiving an individual PGx profile as secondary finding of research

Aims	Themes	Topics

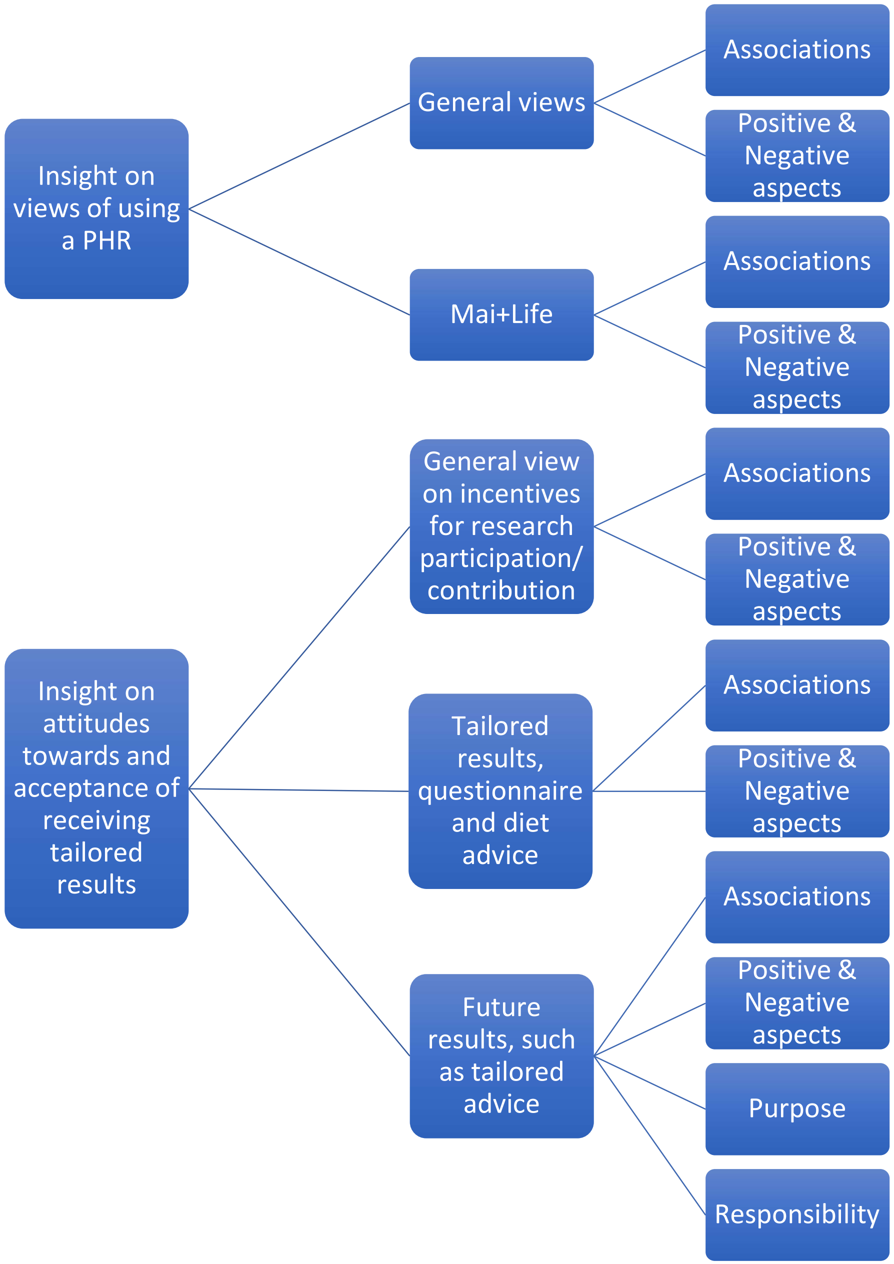

Aims

1. To gain insight in views on use of a Personal Health Record, especially for research (PHR; in Dutch Persoonlijke Gezondheidsomgeving).
2. To understand the attitudes and acceptance of introducing the incentive of return of results.

Themes

1. General views
2. Mai+Life PHR
3. General view on incentives for research participation
4. Tailored diet advice
5. Future tailored advice

Topics

1. Associations PHR
2. Positive & Negative aspects PHR
3. Associations Mai+Life
4. Positive & Negative aspects Mai+Life
5. Associations Incentives for research participation/contribution
6. Associations tailored results, questionnaire and diet advice
7. Positive & Negative aspects tailored results
8. Associations future results of research, such as tailored advice
9. Positive & Negative aspects
10. Purpose of results of research

Dutch working document with example questions - Nederlands Werk document met voorbeeld vragen

1. *Algemene indruk bij gebruik van PGO:* Wat vind je van het gebruiken van een PGO in het algemeen. Wat komt er bij je op?

2. *Positieve & negatieve aspecten PGO algemeen:* Wat zijn algemene positieve aspecten van een PGO? Wat zijn negatieve aspecten van een PGO? Hoe en waarom zijn deze positief en/of negatief voor jou?

3. *Indruk bij gebruik van Mai+Life:* Wat vind je van het gebruiken van een Mai+Life. Wat komt er bij je op?

4. *Positieve & negatieve aspecten gebruik van Mai+Life:* Wat zijn algemene positieve aspecten van een Mai+Life? Wat zijn negatieve aspecten van een Mai+Life? Hoe en waarom zijn deze positief en/of negatief voor jou?

5. *Algemene indruk van het terugkrijgen van resultaten uit onderzoek:* Wat vind je van het terugkrijgen van resultaten uit wetenschappelijk onderzoek?

6. *Algemene indruk van het terugkrijgen van resultaten uit onderzoek:* Wat zijn algemene positieve aspecten van een PGO? Wat zijn negatieve aspecten van een PGO? Hoe en waarom zijn deze positief en/of negatief voor jou?

7. *Indruk Voedingsadvies-op-maat:* Hoe denk je hierover? Wat komt er bij je op?

8. *Positieve & negatieve aspecten Voedingsadvies-op-maat:* Wat zijn algemene positieve aspecten van een PGO? Wat zijn negatieve aspecten van een PGO? Hoe en waarom is dat positief of negatief voor jou?

9. *Toekomstige resultaten, zoals advies op maat:* Wat zijn algemene positieve aspecten van een PGO? Wat zijn negatieve aspecten van een PGO? Hoe en waarom is dat positief of negatief voor jou?

10. *Toekomstige resultaten, zoals advies op maat:* Wat voor doel moeten deze resultaten hebben volgens u?

11. *Toekomstige resultaten, zoals advies op maat:* Wie is er verantwoordelijk voor de resultaten en het gebruik er van volgens u?
