# Supplementary figures and images for: Personal Health Record for Personalizing Research and Care Trajectories: A Proof of Concept Pilot with Diet in Inflammatory Bowel Diseases
Source: J Pers Med. 2023 Mar 29;13(4):601. doi: 10.3390/jpm13040601 (PMC10144383; doi:10.3390/jpm13040601)

## Voedingsadvies

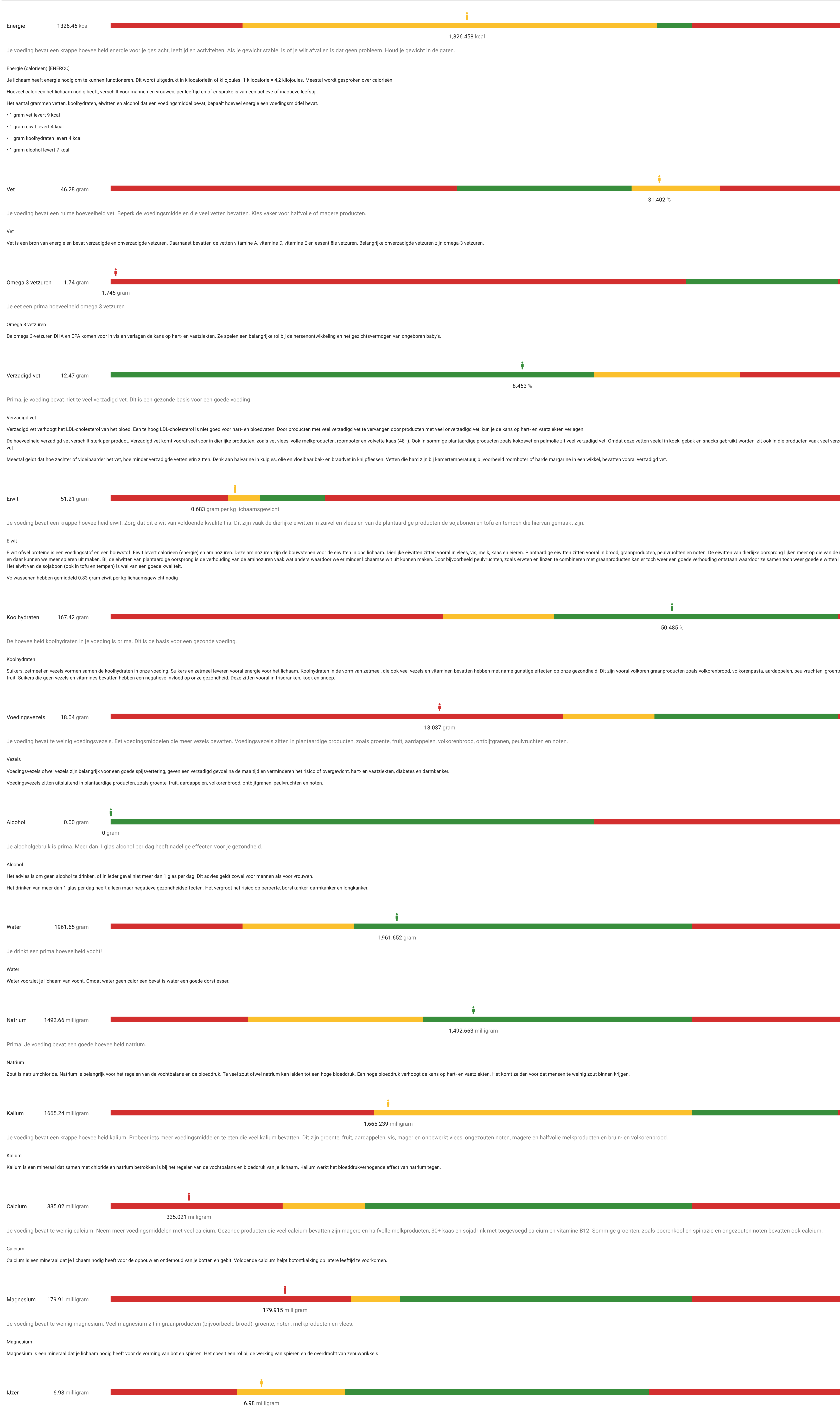

Supplement: Supplementary file 1 [file jpm-13-00601-s001.zip › Mai+Life IBD Pilot Voedingsadvies.pdf]
